# Nitrous Oxide-Induced Myelopathy: A Case From French-Speaking Switzerland

**DOI:** 10.7759/cureus.104698

**Published:** 2026-03-05

**Authors:** Alain Witzig, Jérôme de Massias de Bonne, Camille Galletti, Nicolas Garin

**Affiliations:** 1 Internal Medicine, Hôpital Riviera-Chablais, Rennaz, CHE; 2 Neurology, Hôpital Riviera-Chablais, Rennaz, CHE; 3 Radiology, Hôpital Riviera-Chablais, Rennaz, CHE

**Keywords:** cobalamin deficiency, neurotoxicity, nitrous oxide myelopathy, recreational drug use, sensory ataxia, subacute combined degeneration

## Abstract

Recreational nitrous oxide (N₂O) use has increased worldwide, driven by its accessibility, low cost, and perception as a harmless “party drug.” Chronic or heavy use can cause neurological complications through functional vitamin B₁₂ deficiency. Despite its growing prevalence, such cases remain rarely documented in French-speaking Switzerland. Our patient is a 27-year-old man of Cape Verdean origin who presented with rapidly progressive paresthesia and gait ataxia following heavy N₂O inhalation. Neurological examination revealed profound sensory ataxia and loss of vibration sense in the lower limbs. Laboratory tests showed low serum vitamin B₁₂ with markedly elevated methylmalonic acid and homocysteine levels. Cervical MRI demonstrated a T2-weighted hyperintensity of the dorsal columns (C1-C5) with the characteristic “inverted V” sign, consistent with posterior column myelopathy. Nerve conduction studies were normal, excluding polyneuropathy. Treatment with hydroxocobalamin and methionine supplementation was initiated; folate was started concomitantly rather than after vitamin B₁₂ normalization. At the three-month follow-up, the patient had discontinued vitamin therapy and resumed N₂O use, with persistent sensory deficits and ataxic gait. This case illustrates a typical presentation of N₂O-induced dorsal myelopathy due to functional vitamin B₁₂ deficiency. It represents a rare and probably underreported occurrence in French-speaking Switzerland. Clinicians should maintain high suspicion for N₂O toxicity in young adults presenting with unexplained sensory ataxia. Early recognition, sustained abstinence, and appropriate vitamin B₁₂ replacement are crucial to prevent irreversible neurological damage.

## Introduction

Recreational nitrous oxide (N₂O) use has risen sharply in recent years, facilitated by its low cost, easy accessibility, and general perception as a harmless “party drug.” The European Monitoring Centre for Drugs and Drug Addiction (EMCDDA) identified N₂O in 2022 as one of the most widely used psychoactive substances in Europe [1]. Initially developed for anesthetic use, N₂O is now commonly inhaled from balloons or canisters for its transient euphoric and dissociative effects, which last only a few minutes [2].

Although often considered benign, exposure to N₂O can lead to severe neurological complications, including myelopathy, polyneuropathy, or combined myeloneuropathy, hematological complications such as anemia and thrombosis, and may also cause hypoxemia, respiratory injury, cold-related tissue damage, and psychiatric disturbances [2-4]. The mechanism of neurotoxicity involves oxidation of the cobalt ion within cobalamin, which inactivates methionine synthase in the one-carbon metabolism pathway. This leads to a functional vitamin B₁₂ deficiency, characterized by accumulation of methylmalonic acid and homocysteine, impaired myelin methylation, and subsequent demyelination of the central and peripheral nervous systems [5]. Reducing exposure is currently the only effective way to prevent those neurological and systemic complications, making prevention a key public health priority [1].

Numerous cases have been reported globally, particularly in Europe and Asia [1], but cases from Switzerland, especially in its French-speaking region, remain rare. We report here the first case of N₂O-induced myelopathy diagnosed at Hôpital Riviera-Chablais (Rennaz, Switzerland), a presentation that underscores the need for increased awareness among clinicians in this region.

## Case presentation

A 27-year-old man of West-African origin, employed in construction without formal qualifications, presented to the emergency department of Hôpital Riviera-Chablais on August 28, 2025, with paresthesia in both lower limbs and hands, which had begun 48 hours earlier and had progressively worsened. The sensory symptoms followed a symmetrical and distal pattern and extended up to the pelvis. They were accompanied by worsening unsteadiness and occasional lower-limb weakness without falls.

Neurological examination revealed preserved higher cortical functions. Owing to the patient’s low literacy level and incomplete mastery of the French language, assessment of higher cortical functions was necessarily limited; however, within these constraints, orientation, attention, memory, and language appeared intact. Cranial nerve examination was unremarkable. Motor examination showed normal muscle bulk and tone, with preserved strength graded 5/5 in all four limbs. Sensory examination demonstrated marked sensory ataxia predominantly affecting the lower limbs, with absent vibration sense up to the iliac crests and severely impaired joint position sense at the toes and ankles, while proprioception remained preserved in the upper limbs. Deep tendon reflexes were elicitable within normal reflexogenic zones but were globally decreased, symmetrically, and to a similar degree in both upper and lower limbs (biceps, triceps, brachioradialis, patellar, and Achilles reflexes). Plantar responses were flexor bilaterally, with no Babinski sign. Cerebellar testing revealed no dysdiadochokinesia. The Romberg test was positive, with widening of the base of support. Gait examination showed a profoundly ataxic gait, and tandem walking was impossible. Examination of the skull and spine revealed no abnormalities. Skin hyperpigmentation was not observed. After careful questioning, the patient signaled recent heavy recreational use of N₂O the night preceding symptoms onset. He subsequently disclosed a seven-to-10-year history of N₂O use, occurring two to five times per month and involving five to 20 canisters per episode. He also recalled mild intermittent paresthesia in the same lower-limb distribution over the past two years, for which he had never sought medical attention.

On admission to the ward, he was able to ambulate independently with a pronounced ataxic gait. Physiotherapy for gait re-education and proprioceptive training was initiated.

Laboratory results showed no hematological abnormalities, but vitamin B₁₂ was low, while holotranscobalamin was elevated. Thiamine was elevated, methylmalonic acid was markedly increased, and total homocysteine was significantly elevated. Detailed laboratory values are presented in Table 1.

**Table 1:** Laboratory findings at presentation

Laboratory parameter	Result	Reference range
Leucocytes	6.9 G/L	4.0–10.0 G/L
Erythrocytes	4.82 T/L	4.40–5.90 T/L
Hemoglobin	140 g/L	135–170 g/L
Hematocrit	0.43 L/L	0.40–0.52 L/L
Mean corpuscular volume	89 fL	80–100 fL
Mean corpuscular hemoglobin concentration	329 g/L	310–360 g/L
Thrombocytes	300 G/L	150–350 G/L
Vitamin B₁₂	139 pmol/L	145–569 pmol/L
Holotranscobalamin	>300 pmol/L	37.5–188 pmol/L
Thiamine (vitamin B₁)	153 nmol/L	78–143 nmol/L
Methylmalonic acid	4.94 µmol/L	<0.28 µmol/L
Total homocysteine	54.4 µmol/L	5.0–15.0 µmol/L
Albumin-corrected total calcium	2.32 mmol/L	2.10–2.55 mmol/L
Magnesium	0.83 mmol/L	0.70–1.10 mmol/L
Phosphate	1.34 mmol/L	0.87–1.45 mmol/L
Ferritine	168 µmol/L	30–400 µmol/L
CRP	< 14.6 mg/L	<5 mg/L
Erythrocyte sedimentation rate	7 mm/h	<15 mm/h
ALAT	12 U/L	<50 U/L
ASAT	17 U/L	<50 U/L
Albumine	41.8 g/L	35.0–52.0 g/L
Thyroid-stimulating hormone	1.25 mUI/L	0.27–4.20 mUI/L
Creatinine	85 µmol/L	<106 µmol/L
Glomerular filtration rate (CKD-EPI 2009)	108 mL/min/1.73m2	>60 mL/min/1.73 m^2^

**Figure 1:**
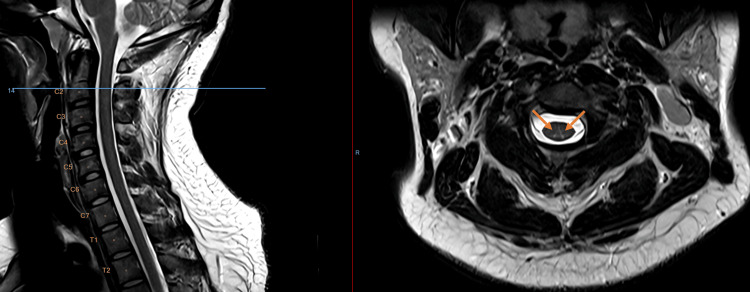
Axial T2-weighted MRI (3T) imaging of the cervical spine (C1-2) Linear T2 hyperintensities of the dorsal columns of the cervical spine forming an “inverted V sign” (orange arrows), characteristic of functional vitamin B₁₂ deficiency caused by N₂O toxicity, extending from C1 to C5. The hyperintensities are not visible on the sagittal plane because of spatial resolution.

**Figure 2:**
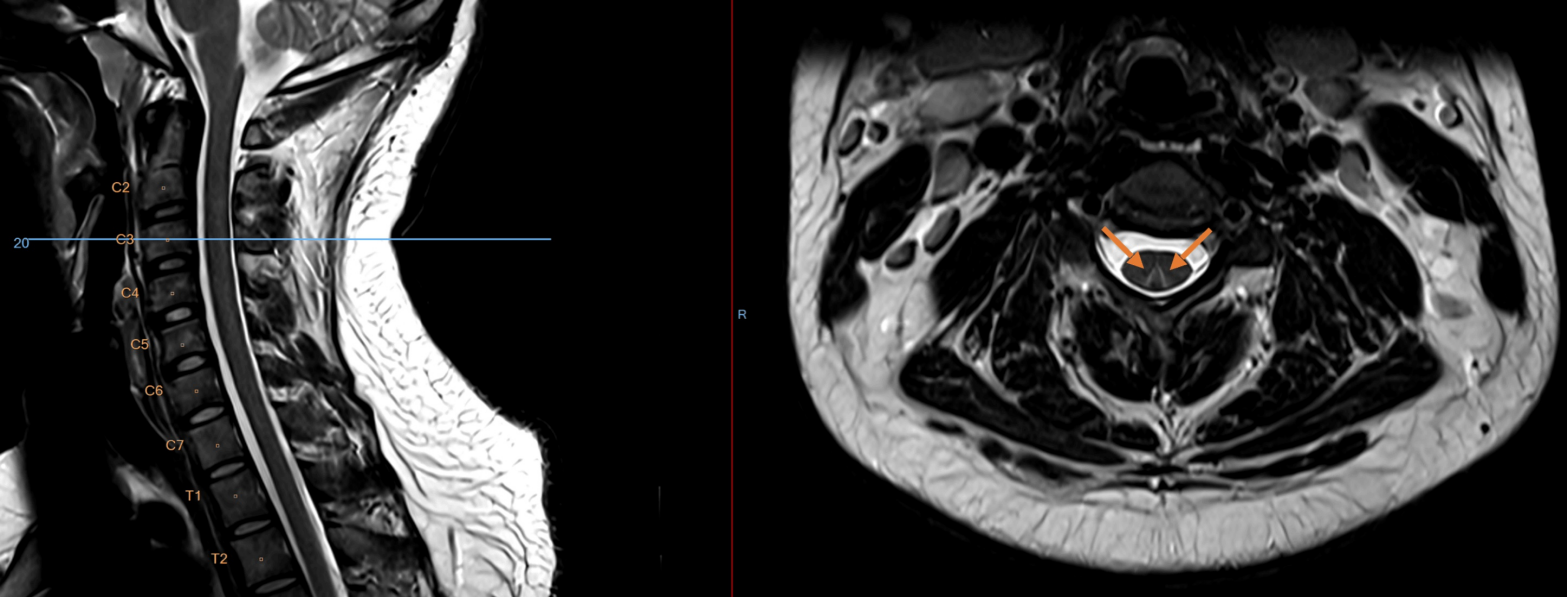
Axial T2-weighted MRI (3T) imaging of the cervical spine (C3)

**Figure 3:**
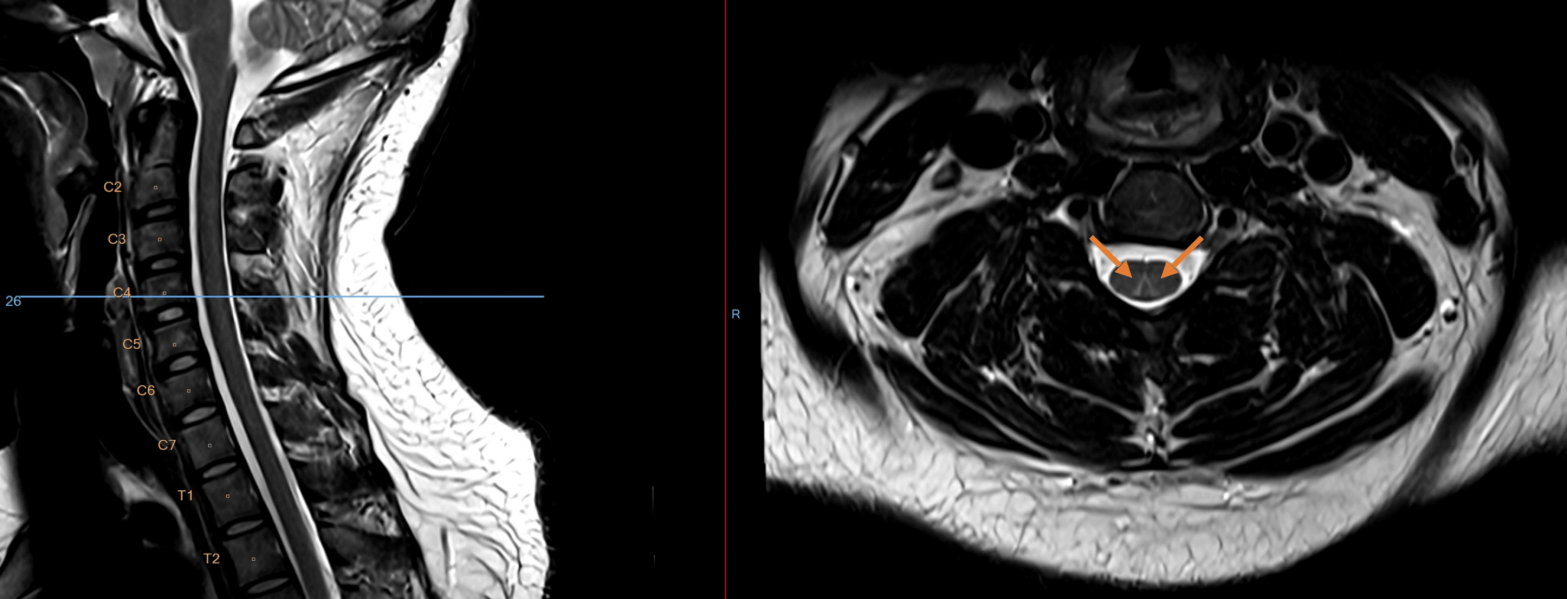
Axial T2-weighted MRI (3T) imaging of the cervical spine (C4)

**Figure 4:**
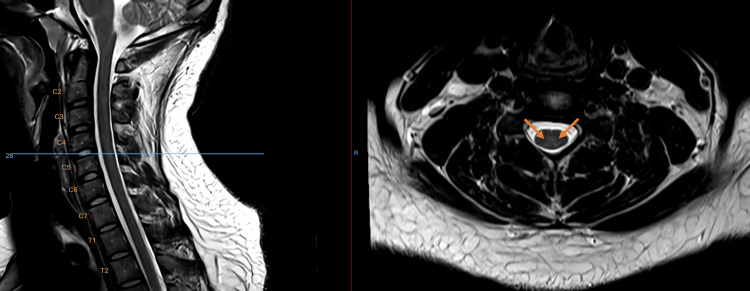
Axial T2-weighted MRI (3T) imaging of the cervical spine (C4-5)

Magnetic resonance imaging (MRI) of the cervical spine demonstrated a T2-weighted hyperintensity of the dorsal columns extending from C1 to C5, with a characteristic “inverted V” pattern on axial images (Figures 1-4). While this pattern is not specific, in the present clinical context, it is consistent with N₂O-induced posterior column myelopathy.

Treatment was initiated with hydroxocobalamin 1 mg subcutaneously daily for seven days, followed by oral cobalamin 2 mg daily until resolution of symptoms. Methionine supplementation (1 g three times daily) was started for six weeks, as well as folate supplementation once vitamin B₁₂ therapy was initiated.

Electrophysiological studies performed on August 28, 2025, revealed normal segmental motor conduction in the right ulnar, right common peroneal, and left tibial nerves. F-wave latencies were not prolonged in any of these three nerves, with F-waves being inconstant in the peroneal nerve, which is considered a physiological finding. Sensory antidromic conduction studies of the right ulnar and left sural nerves showed entirely normal conduction parameters. Electromyography of the right tibialis anterior muscle did not reveal signs of acute or chronic denervation. Nevertheless, in the context of an acute presentation, electromyography alone is insufficient to formally exclude polyneuropathy. Tables 2-5 detail the electrophysiological values obtained for each of the nerves mentioned above. With regard to needle electromyography, the findings are reported in narrative form only.

**Table 2:** Motor nerve conduction studies

Nerve	Recording muscle	Stimulation site	Distal latency (ms)	Compound muscle action potential amplitude (mV)	Distance (mm)	Conduction velocity (m/s)
Right ulnar nerve	Abductor digiti minimi	Wrist	2.8	9.0	70	-
		Below elbow	6.6	8.2	215	57
		Above elbow	8.3	8.2	100	62
Right common peroneal nerve	Extensor digitorum brevis	Ankle	4.7	5.4	100	-
		Fibular head	10.9	5.1	310	50
		Popliteal fossa	12.7	5.1	90	50
Right tibial nerve	Abductor hallucis	Ankle	5.1	7.0	-	-
		Popliteal fossa	14.0	5.0	415	47

**Table 3:** Late spinal responses (F waves)

Nerve	Recording muscle	Stimulation site	Minimum F-wave latency (ms)	Maximum F-wave latency (ms)	Persistence (%)
Right ulnar nerve	Abductor digiti minimi	Wrist	27.7	37.1	53.8
Right common peroneal nerve	Extensor digitorum brevis	Ankle	47.9	50.1	20
Right tibial nerve	Abductor hallucis	Ankle	48.8	61.4	63.6

**Table 4:** Sensory nerve conduction studies

Nerve	Recording muscle	Stimulation site	Onset latency (ms)	Sensory nerve action potential amplitude (µv)	Distance (mm)	Conduction velocity (m/s)
Right ulnar nerve	Fifth digit	Wrist (antidromic)	3.4	14.3	120	46
Right sural nerve	Lateral malleolus	Calf (antidromic)	2.5	11.8	95	45

**Table 5:** Electromyography summary

Muscle	Spontaneous activity			Volitional motor unit action potential					Maximum volitional activity		
Right tibialis anterior	Fibrillation potentials	Positive Sharp Waves	Fasciculations	Duration	Amplitude	Polyphasia	Motor Unit Configuration	Recruitment	Amplitude	Interference pattern	Effort
	None	None	None	Normal	Normal	None	Normal	Normal	Normal	Full	Maximal

Based on the clinical, anamnestic, radiological, electrophysiological, and laboratory profile, a diagnosis of dorsal myelopathy secondary to functional vitamin B₁₂ deficiency induced by repeated recreational N₂O use was made. In this highly suggestive context, further investigations for alternative differential diagnoses were not pursued; these are discussed in the Discussion section.

Three months later, at a phone follow-up, the patient declared that he had not adhered to the prescribed vitamin B₁₂ treatment and continued N₂O consumption, a statement that was confirmed by his general practitioner. He reported persistent paresthesia extending to the knees and a markedly ataxic gait, suggesting that he had irreversible neurological sequelae secondary to lack of abstinence and adherence to therapy.

## Discussion

Recreational use of N₂O has evolved into a major public health concern, especially among young adults. International surveys have estimated a lifetime prevalence of 17% and annual use of 11.9%, with users being mostly men (69.3%), 4.2% of users reporting persistent sensory symptoms in a stocking-glove distribution. Men reported paraesthesia more frequently at lower doses, but at higher doses, women demonstrated a greater predicted probability of symptoms, suggesting possible sex-related differences. Easy availability, low price, and lack of strict regulation have fostered its rapid spread. Despite this, awareness of its neurological toxicity remains limited among healthcare professionals [6].

**Figure 5:**
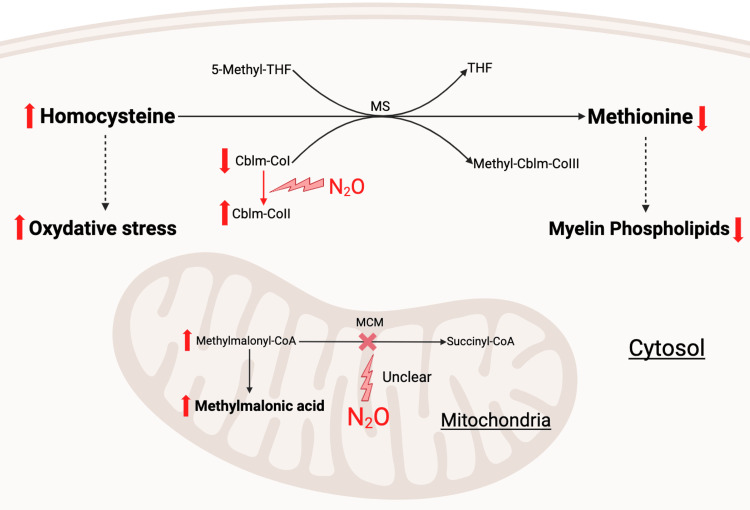
Impact of N2O on metabolism Cblm: cobalamin, N_2_O: nitrous oxide, MCM: methylmalonyl-CoA-mutase, MMA: methylmalonic acid, MS: methionine synthase, THF: tetrahydrofolate Image created by the authors with BioRender (All rights reserved)

N₂O exerts its neurotoxic effects by oxidizing the cobalt ion in vitamin B₁₂, thereby inactivating methionine synthase and methylmalonyl-CoA mutase [2]. This disruption impairs methionine and S-adenosylmethionine synthesis, both of which are essential for myelin phospholipid methylation. The resulting functional vitamin B₁₂ deficiency disrupts myelin methylation, leading to demyelination of the dorsal and lateral columns of the spinal cord, particularly in the cervical region. Oxidative stress and NMDA receptor-mediated excitotoxicity may contribute further, as well as a dysregulation of cytokines and growth factors that regulate myelin integrity [7]. Approximately 30% of affected individuals display normal B₁₂ values, underscoring the diagnostic importance of elevated homocysteine and methylmalonic acid, both of which increased in over 90% of cases [7]. Figure 5 schematically illustrates the effect of N₂O on metabolic pathways.

Elevated homocysteine contributes not only to demyelination but also to vascular complications, including deep vein thrombosis and pulmonary embolism [3]. However, as homocysteine levels normalize within several days after cessation of exposure, diagnostic sensitivity declines rapidly over time [7].

The neurological manifestations of N₂O toxicity are variable, ranging from chronic sensory length-dependent polyneuropathy to subacute predominant length-dependent axonal polyneuropathy or acute polyradiculoneuropathy [4]. Moreover, 96% of patients present with sensory deficits, unsteady gait, and/or weakness, often accompanied by alternating hypo- and hyperreflexia depending on the predominance of peripheral or central involvement. This mixed picture has been described as myeloneuropathy [3]. Electrophysiological studies can demonstrate mixed axonal and demyelinating neuropathies, though they may also remain normal [2], as in our patient.

Acute complications of N₂O use may include altered cognition, hypoxemia, respiratory irritation with potential development of interstitial emphysema or pneumomediastinum, cold-related injuries (notably involving the oral cavity or hands), and, in rare cases, death due to asphyxia. Chronic exposure has been associated with neuropsychiatric manifestations such as encephalopathy, behavioral changes, paranoia, delusions, hallucinations, and other psychiatric symptoms, as well as hematological and systemic complications, including anemia, skin hyperpigmentation, and thrombosis, the latter being rarely reported [3,4].

Neuroimaging with MRI often shows T2-weighted hyperintensities of the dorsal columns in the cervical spinal cord, forming an “inverted V” or “rabbit ear” pattern, particularly between C3 and C5 [2]. This pattern reflects posterior column involvement, a characteristic feature of N₂O-induced myelopathy, although normal MRI findings do not rule out the diagnosis. These lesions reflect demyelination of the posterior columns but may be absent in up to one-third of cases, especially after prolonged exposure, likely due to resolution of edema [8]. Posterior column hyperintensity on spinal MRI is a non-specific finding and may be observed in several other conditions affecting the dorsal columns. Differential diagnoses to consider include copper deficiency myelopathy, subacute combined degeneration secondary to vitamin B12 deficiency, N₂O-induced myelopathy, HIV-associated vacuolar myelopathy, hereditary spastic paraplegia, and multiple sclerosis. Metabolic and toxic causes, such as folate-related myelopathy, as well as vascular etiologies including posterior spinal artery ischemia leading to spinal cord infarction, can also be considered. Less frequent causes such as neurosyphilis, vitamin E deficiency, and paraneoplastic or inflammatory myelopathies may present with similar imaging features [9].

The differential diagnosis of acute or subacute ataxic polyneuropathy includes infectious (e.g., HIV, Lyme disease, hepatitis, leprosy), metabolic (porphyria and deficiencies of vitamins B1, B6, B9, and B12), toxic and drug-induced causes (e.g., N₂O, heavy metals, cisplatin, ethambutol, amiodarone), autoimmune conditions (e.g., Guillain-Barré syndrome, Sjögren’s syndrome, vasculitis, sarcoidosis), as well as hereditary and paraneoplastic neuropathies [10]. In the present case, further evaluation for these alternative etiologies was not pursued, as the clinical presentation, exposure history, laboratory findings, neuroimaging, and temporal profile were all highly consistent with N₂O-induced neurotoxicity.

Treatment requires immediate cessation of N₂O exposure and prompt initiation of vitamin B₁₂ replacement. Hydroxocobalamin should be administered 1 mg subcutaneously or intramuscularly once daily for approximately two weeks, followed by high-dose oral supplementation (2 mg daily) until complete clinical recovery. Adjunctive methionine supplementation may enhance remyelination and restore methylation balance, and is prescribed as a 1 g oral dose three times per day for a minimum of 4 to 6 weeks, or until substantial symptomatic improvement occurs. Because folate administration prior to vitamin B₁₂ repletion may exacerbate neurological dysfunction, current recommendations advise delaying folate supplementation until vitamin B₁₂ levels have normalized [11]. However, in the presented case, folate therapy was initiated concomitantly with vitamin B₁₂ replacement, before reassessment of cobalamin levels, which represents a deviation from standard management protocols. With appropriate treatment and strict abstinence, most patients show substantial neurological recovery within weeks to months, although up to one-third experience persistent sensory disturbances or gait instability, particularly in cases of delayed recognition or ongoing N₂O use [11]. Our patient belongs to this latter group, having discontinued vitamin supplementation and resumed N₂O consumption, with persistent lower-limb paresthesia and ataxic gait at three months.

Overall, the clinical, radiological, and biological features observed in our patient are largely consistent with previously published data on N₂O-related neurotoxicity [1,3]. The patient’s young age, long-standing recreational N₂O exposure, predominant sensory symptoms with gait instability, and isolated posterior column involvement on MRI closely mirror the most frequently reported manifestations in the literature. In addition, the presence of a functional vitamin B₁₂ deficiency with markedly elevated methylmalonic acid and homocysteine levels aligns with the metabolic profile described in the majority of reported cases. By contrast, the absence of hematological abnormalities and the normal electrophysiological studies represent less common findings, as anemia and peripheral neuropathy are frequently reported in published series. Furthermore, the relatively acute onset of symptoms differs from the more typical subacute presentation. Together, these findings highlight both the characteristic features of N₂O-induced myelopathy and the clinical heterogeneity of this condition.

In Switzerland, the prevalence of recreational N₂O use remains poorly documented and appears relatively marginal at the population level. According to Infodrog, 30-day self-reported use increased from 5% in 2022 to 8% in 2023, while 12-month prevalence declined from 13% to 11%, arguing against a clear overall rise in consumption; current public health measures therefore prioritize prevention and risk awareness over strict legal restrictions, and the Federal Council considers tighter regulation or prohibition to be disproportionate at present [12]. In this context, the present case represents, to our knowledge, the first identified at our hospital, and a retrospective review at Lausanne University Hospital (CHUV), the cantonal university hospital of the Canton of Vaud, identified only one comparable case over the past 20 years.

## Conclusions

This case represents a rare and likely underreported occurrence of N₂O-induced myelopathy in French-speaking Switzerland, emphasizing the need for greater clinical awareness. It illustrates the need for increased clinical awareness and early recognition of this reversible but potentially disabling condition. Because the symptoms are subtle and may mimic other causes of sensory ataxia, detailed history taking, especially regarding recreational drug use, is critical. Public health interventions and education targeting young adults are equally necessary to curb this preventable form of neurotoxicity.
